# The effectiveness of art therapy on motor function in Parkinson’s disease: a systematic review and meta-analysis

**DOI:** 10.3389/fpsyg.2025.1542405

**Published:** 2025-09-04

**Authors:** Zexi Liu, Chen Hui, Shuhan Liu, Yujia Chen, Yihan Zhang, Wenliang Ye

**Affiliations:** ^1^Nanhai College of Arts and Technology, Haikou University of Economics, Haikou, China; ^2^College of arts, Chonbuk National University, Jeonju, Republic of Korea; ^3^Zhangjiajie Hospital of Traditional Chinese Medicine, Zhangjiajie, China; ^4^Department of International Cultural Education, Chodang University, Muan, Republic of Korea

**Keywords:** Parkinson’s disease, art therapy, dance therapy, gait, motor function, meta-analysis

## Abstract

**Objective:**

This meta-analysis aimed to evaluate the effectiveness of art therapy interventions in improving motor function performance in patients with Parkinson’s Disease (PD), with a focus on identifying the most effective modalities.

**Method:**

Randomized controlled trials were identified through searches in PubMed, Embase, Web of Science, and Cochrane Library. Twenty-six studies were included, assessed for quality, and analyzed following PRISMA guidelines (PROSPERO: CRD42024611770). Subgroup analyses were performed for primary outcomes (UPDRS, TUG, Mini−BESTest), while secondary outcomes (Stride Length, FOG, 6MWT, and Gait Speed) were evaluated using forest and funnel plots to estimate pooled effects.

**Results:**

Art therapy significantly improved motor function, as evidenced by reductions in UPDRS III scores (SMD = −0.44, 95% CI [−0.61, −0.26], *p* < 0.05), TUG scores (SMD = −0.25, 95% CI [−0.41, −0.10], *p* < 0.05), and increases in Mini-BESTest scores (SMD = 0.41, 95% CI [0.10, 0.72], *p* < 0.05). Among the interventions, dance therapy demonstrated the most significant effects on motor function (UPDRS III: SMD = −0.52, 95% CI [−0.78, −0.26], *p* < 0.05; TUG: SMD = −0.37, 95% CI [−0.58, −0.17], *p* < 0.05; Mini-BESTest: SMD = 0.56, 95% CI [0.25, 0.87], *p* < 0.05). Secondary outcomes revealed small to moderate improvements in gait speed (SMD = 0.34, *p* < 0.05), 6MWT (SMD = 0.41, *p* < 0.05), FOG (SMD = −0.33, *p* < 0.05), and stride length (SMD = 0.59, *p* < 0.05). Although the findings were robust, high heterogeneity in certain outcomes highlights the need for standardized intervention protocols to ensure consistency and reproducibility.

**Conclusion:**

This study underscores the clinical significance of art therapy in improving motor functions in PD patients. Among the interventions, dance therapy exhibited the most pronounced effects, highlighting its potential as a pivotal component in multidisciplinary neurorehabilitation programs.

**Systematic Review Registration:**

https://www.crd.york.ac.uk/prospero/display_record.php?ID=CRD42024611770, identifier (CRD42024611770).

## 1 Introduction

The disability and mortality rates of PD (PD), an incurable progressive neurodegenerative disorder, are rising faster than those of any other neurological condition (Playfer, 1997; World Health Organization, n.d.). Early loss of dopaminergic neurons in the substantia nigra pars compacta (SNpc) leads to dopamine deficiency in the basal ganglia and dysfunction in the basal ganglia-supplementary motor area (BG-SMA) interaction (Cunnington et al., 1996), resulting in movement disorders characterized by classic Parkinsonian motor symptoms (Kalia and Lang, 2015). These motor symptoms include resting tremors, bradykinesia, rigidity, postural instability, freezing of gait, and balance difficulties (Kuhlman et al., 2019; Morris et al., 1994). Motor decline also directly affects non-motor functions, such as depression, anxiety, obsessive-compulsive behaviors, cognitive decline, reduced quality of life, and autonomic dysfunction (e.g., bladder and sexual issues) (Chaudhuri and Schapira, 2009; Postuma and Montplaisir, 2009). The diagnosis of PD relies on clinical motor signs as key indicators (Postuma et al., 2015), Reduction in the amplitude of step size (hypokinesia) is a regular feature of PD (Morris et al., 1994) and gait disturbances are closely associated with disease severity (Iansek et al., 2006). Gait disturbances and impaired motor function are major contributors to the decline in quality of life and loss of independence among PD patients, profoundly impacting their physical and mental health.

Dopaminergic medications are the standard treatment for PD and are effective in managing motor symptoms during early stages (Cotzias et al., 1969), however, they often fail to address the full spectrum of motor symptoms (Fahn, 2008; Bohannon, 1993). Over time, complications masked by these medications may emerge (Lang and Lozano, 1998). The progressive worsening of PD symptoms, combined with the high costs of traditional treatments (Balestrino and Schapira, 2020), underscores the urgent need for adjunctive interventions that are cost-effective, safe, and effective in clinical practice.

Art therapy is an umbrella term for disciplines that use creative expression within therapeutic relationships to facilitate emotional exploration, self-awareness, and healing. This heterogeneous field encompasses distinct modalities: visual art therapy employs non-verbal expression through painting or sculpting to process emotions; dance/movement therapy integrates rhythmic auditory cues and body awareness to enhance motor-cognitive connections; music therapy uses structured sound patterns for neurological retraining; and drama therapy focuses on role-play for interpersonal skill development (Kaplan, 2003). As a safe and reliable adjunctive intervention, art therapy has demonstrated effectiveness across clinical contexts including stroke rehabilitation (Kongkasuwan et al., 2016), cancer care (Boehm et al., 2014), mental health (Moo and Ho, 2024; Zhang et al., 2024), and diabetes management (Krishnan et al., 2015; Yang et al., 2021). The current review specifically examines movement-based creative interventions (dance therapies) within Parkinson’s disease (PD) care, where their multisensory integration mechanisms—combining rhythmic auditory stimulation, visual prompts, and choreographed movements—synergize with social engagement to target motor dysfunction. Culturally specific approaches, such as Biodanza, tango, and Qi dance, enhance gait rhythm through rhythmic auditory stimulation and improve adherence through social engagement. Studies suggest these methods positively impact gait speed, stride length, freezing of gait, motor function, attention, and cognition (Morris et al., 1994).

Despite several clinical trials, no comprehensive meta-analysis has evaluated the effects of art therapy on physical function and gait in PD. This study addresses this gap by providing evidence-based insights to support cost-effective, personalized, non-pharmacological interventions for clinical practice and research. In this study, “body function” is defined as a multidimensional concept encompassing both gait and dynamic balance, with gait being a core component.

## 2 Methods

The study strictly follows the Preferred Reporting Items for Systematic Reviews and Meta-Analyses (PRISMA) reporting checklist and has been registered in PROSPERO (CRD42024611770). Subgroup analyses were performed exclusively for primary outcomes (UPDRS III, TUG and Mini-BESTest), which are central to evaluating motor function in PD. Secondary outcomes (Stride Length, FOG, 6MWT, and Gait Speed) were quantitatively analyzed using forest plots and funnel Plots to estimate pooled effects.

### 2.1 Search strategy

A thorough literature search was performed across multiple electronic databases, including the Cochrane Library, Web of Science, PubMed, and EMBASE, covering publications from their inception up to July 31, 2024. Using a comprehensive search strategy that combined Medical Subject Headings (MeSH) and free-text terms, we screened titles, abstracts, keywords, and subsequently conducted a full-text review. The temporal range of this review spanned from January 1900 to July 2024, with non-English language literature excluded. These time and language restrictions were applied to focus the research team on the latest, relevant studies within their language proficiency, thereby enhancing the quality and validity of the findings. The search strategy, initially based on PubMed, was extended to additional databases. A manual search was also conducted by reviewing the bibliographies of pertinent articles and references within the selected studies.

The detailed search formula was as follows:

((“1900/01/01”[Date - Publication]: “2024/07/31”[Date - Publication])) AND (((((trial[Title/Abstract]) OR (random*[Title/Abstract])) OR (“Randomized Controlled Trial” [Publication Type])) AND ((((((“singing”[Title/Abstract] OR “dancing”[Title/Abstract] OR “painting”[Title/Abstract] OR “drawing”[Title/Abstract] OR “calligraphy”[Title/Abstract] OR “music”[Title/Abstract] OR “sculpture”[Title/Abstract] OR “collage”[Title/Abstract] OR “poetry”[Title/Abstract] OR “drama”[Title/Abstract] OR “clay”[Title/Abstract] OR “theater”[Title/Abstract]) OR (“visual art therapy”[Title/Abstract])) OR (“yoga”[Title/Abstract])) OR (“mindfulness”[Title/Abstract])) OR (“meditation therapy”[Title/Abstract])) OR (“Art Therapy”[Mesh]))) AND ((“Parkinson Disease”[Mesh]) OR ((((((((((((Idiopathic Parkinson’s Disease[Title/Abstract]) OR (Lewy Body Parkinson’s Disease[Title/Abstract])) OR (Parkinson’s Disease, Idiopathic[Title/Abstract])) OR (Parkinson’s Disease, Lewy Body[Title/Abstract])) OR (Parkinson Disease, Idiopathic[Title/Abstract])) OR (Parkinson’s Disease[Title/Abstract])) OR (Idiopathic Parkinson Disease[Title/Abstract])) OR (Lewy Body Parkinson Disease[Title/Abstract])) OR (Primary Parkinsonism[Title/Abstract])) OR (Parkinsonism, Primary[Title/Abstract])) OR (Paralysis Agitans[Title/Abstract])) OR (Parkinson[Title/Abstract])))). Additionally, a manual search was conducted through the bibliographies of relevant review articles and studies cited in the selected papers. The full search strategies for each database are documented in Table 1.

**Table 1:** Search strategy on PubMed.

Number	Search terms
#1	Parkinson disease [MeSH]
#2	Idiopathic Parkinson’s disease [Title/Abstract]
#3	Lewy body Parkinson’s disease [Title/Abstract]
#4	Parkinson’s disease idiopathic [Title/Abstract]
#5	Parkinson’s disease Lewy body [Title/Abstract]
#6	Parkinson disease idiopathic [Title/Abstract]
#7	Parkinsons disease [Title/Abstract]
#8	Idiopathic Parkinson disease [Title/Abstract]
#9	Lewy body Parkinson disease [Title/Abstract]
#10	Primary parkinsonism [Title/Abstract]
#11	Parkinsonism primary [Title/Abstract]
#12	Paralysis agitans [Title/Abstract]
#13	#2 OR #3 OR #4 OR #5 OR #6 OR #7 OR #8 OR #9 OR #10 OR #11 OR #12 OR #13
#14	#1 OR #2 OR #3 OR #4 OR #5 OR #6 OR #7 OR #8 OR #9 OR #10 OR #11 OR #12 OR #13
#15	Art therapy [MeSH]
#16	Singing [Title/Abstract]
#17	“Dancing” [Title/Abstract]
#18	“Painting” [Title/Abstract]
#19	“Drawing” [Title/Abstract]
#20	“Calligraphy” [Title/Abstract]
#21	“Music” [Title/Abstract]
#22	“Sculpture” [Title/Abstract]
#23	“Collage” [Title/Abstract]
#24	“Poetry” [Title/Abstract]
#25	“Drama” [Title/Abstract]
#26	Theater [Title/Abstract]
#27	“Clay” [Title/Abstract]
#28	Visual arts therapy [Title/Abstract]
#29	Meditation therapy [Title/Abstract]
#30	Mindfulness [Title/Abstract]
#31	Yoga [Title/Abstract]
#32	Psychodrama [Title/Abstract]
#33	#16 OR #17 OR #18 OR #19 OR #20 OR #21 OR #22 OR #23 OR #24 OR #25 OR #26 OR #27 OR #28 OR #29 OR #30 OR #31 OR #32
#34	#15 OR#16 OR #17 OR #18 OR #19 OR #20 OR #21 OR #22 OR #23 OR #24 OR #25 OR #26 OR #27 OR #28 OR #29 OR #30 OR #31 OR #32
#35	“Randomized controlled trial” [Publication Type]
#36	“Trial” [Title/Abstract]
#37	“random*” [Title/Abstract]
#38	“Randomized controlled trial” [Publication Type]
#39	#35 OR #36 OR #37 OR #38
#40	#14 OR #34 OR #39

MeSH, Medical Subject Headings.

### 2.2 Inclusion and exclusion criteria

#### 2.2.1 Inclusion criteria

The selection process follows the PICOS framework (Population, Intervention, Comparison, Outcome, and Study Design) to ensure consistency and rigor:

**Population**: (1) Individuals clinically diagnosed with PD by qualified clinicians or established diagnostic guidelines. (2) Aged ≥ 40 years, capable of standing for at least 30 min, and able to walk independently for a minimum of 3 meters (with or without assistive devices). (3) Excludes participants with psychiatric conditions other than PD or those unable to continue observation due to severe physical illness.

**Intervention**: (1) Experimental group: Any form of art therapy (e.g., singing, dancing, painting, yoga, or other artistic expressions), including combined therapies. (2) Control group: Standard care, no intervention, or non-art-based therapies.

**Outcome**: (1) Primary outcome measures include UPDRS III, TUG, Ministerial. (2) Secondary outcomes include 6MWT, Stride Length, FOG, and Gait Speed.

**Study design**: (1) Only randomized controlled trials (RCTs) published in English and reporting original clinical data. (2) Excludes case reports, editorials, reviews, conference abstracts, and other non-clinical formats.

#### 2.2.2 Exclusion criteria

1. Literature with titles and abstracts in English (or American English) but with the full text in a different language will not be considered.
2. Non-randomized studies, such as reviews, animal studies, study protocols, conference abstracts, case reports, and online reports, are ineligible.
3. Studies lacking complete experimental data or having missing information will not be included.
4. Observational studies will be excluded.

Overall, the inclusion criteria for the meta-analysis are designed to include only high-quality and rigorously conducted studies.

### 2.3 Study selection and data extraction

Records that align with the specified search criteria will be imported into Endnote 20 for deduplication purposes. Two independently evaluated the relevance of search results and extracted data using a standardized form, an initial review was conducted, and any differences between the reviewers were discussed until a consensus was reached. If an agreement could not be achieved, a third reviewer was consulted to make a final decision after discussions with the original two reviewers. To measure the agreement between the two primary reviewers during abstract and full-text screening, kappa values were calculated following established guidelines. Prioritizing intention−to−treat data over completer analysis data when available. They recorded details from each article, such as publication year, study location, diagnostic criteria, sample size, mean/median age, percentage of male participants, registration number, intervention method, and outcomes of interest. For studies with missing or unextractable data, attempts were made to contact the authors for additional information.

### 2.4 Quality assessment

**Figure 1:**
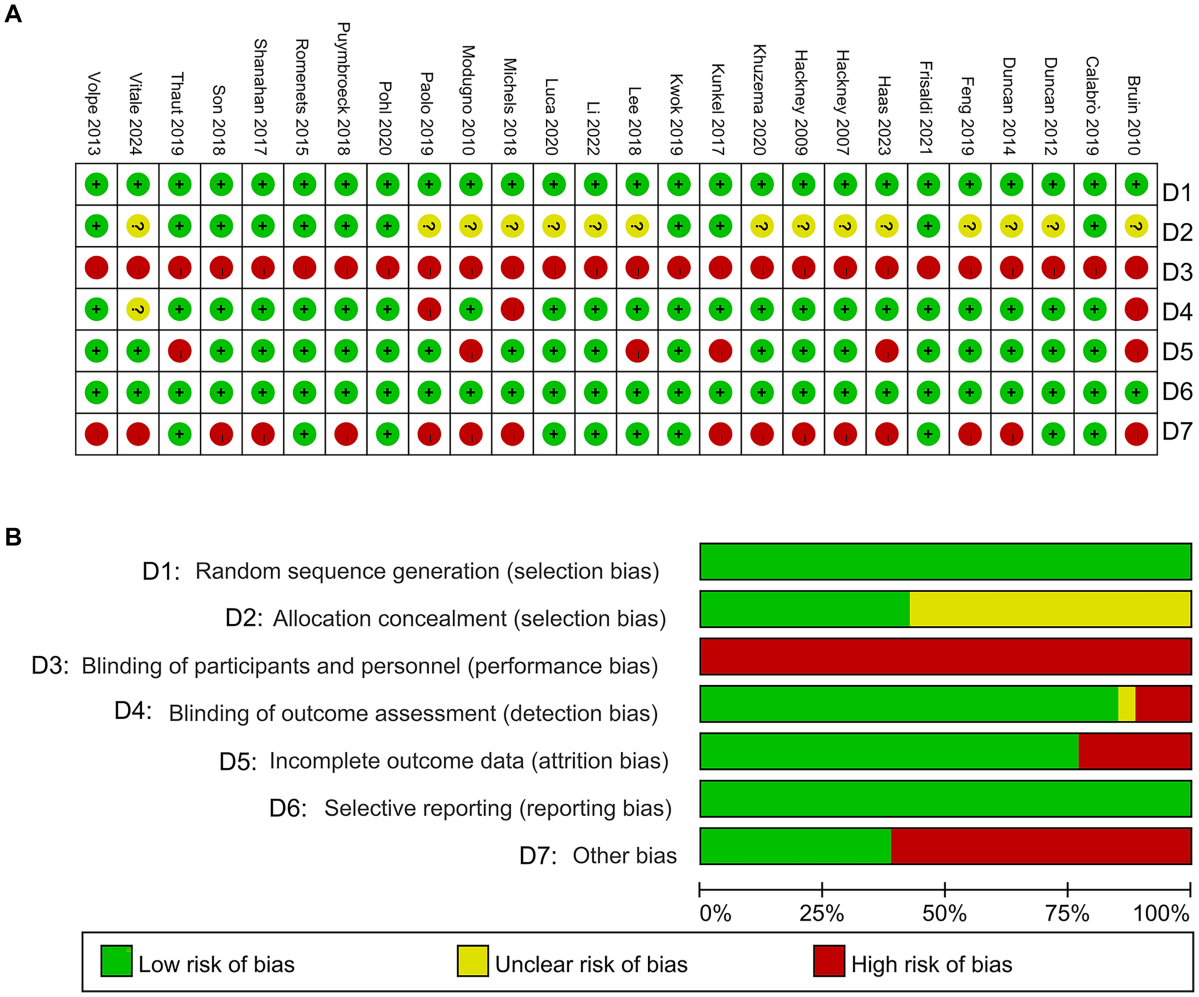
**(A)** Methodological quality assessment of included studies. **(B)** Distribution of methodological quality across included studies.

The methodological quality of the included studies was assessed using the Cochrane Risk of Bias Tool 2.0. This tool evaluates key domains, including randomization process, deviations from intended interventions, missing outcome data, measurement of outcomes, and selection of reported results, ensuring a comprehensive assessment of potential bias. Two independent reviewers conducted the evaluations, and any discrepancies were discussed to reach consensus. If consensus was not achievable, a third reviewer provided the final decision. This rigorous quality assessment establishes a robust foundation for the meta-analysis, enhancing the validity and reliability of the findings. Which examines the generation of random sequences, allocation concealment, blind method for implementers and participants, blinding of outcome assessors, incomplete data, selective reporting, and other sources of bias. Studies were classified as “low risk,” “unclear,” or “high risk” of bias (Figure 1).

D1: For random sequence generation, 26 studies (Volpe et al., 2013; Vitale et al., 2024; Van Puymbroeck et al., 2018; Thaut et al., 2019; Son and Choi, 2018; Solla et al., 2019; Shanahan et al., 2017; Romenets et al., 2015; Pohl et al., 2020; Modugno et al., 2010; Michels et al., 2018; Li et al., 2022; Lee et al., 2018; Kwok et al., 2019; Kunkel et al., 2017; Khuzema et al., 2020; Hackney et al., 2007; Hackney and Earhart, 2009; Haas et al., 2024; Frisaldi et al., 2021; Feng et al., 2019; Duncan and Earhart, 2014; Duncan and Earhart, 2012; De Luca et al., 2020; de Bruin et al., 2010; Calabrò et al., 2019) demonstrated a low risk of bias, accounting for 100%.

D2: Concerning allocation concealment, 11 studies (Volpe et al., 2013; Van Puymbroeck et al., 2018; Thaut et al., 2019; Son and Choi, 2018; Shanahan et al., 2017; Romenets et al., 2015; Pohl et al., 2020; Kwok et al., 2019; Kunkel et al., 2017; Frisaldi et al., 2021; Calabrò et al., 2019) were found to have a low risk of bias (42.31%), while the remaining 57.69% had an unclear risk.

D3: 28 studies (Volpe et al., 2013; Vitale et al., 2024; Van Puymbroeck et al., 2018; Thaut et al., 2019; Son and Choi, 2018; Solla et al., 2019; Shanahan et al., 2017; Romenets et al., 2015; Pohl et al., 2020; Modugno et al., 2010; Michels et al., 2018; Li et al., 2022; Lee et al., 2018; Kwok et al., 2019; Kunkel et al., 2017; Khuzema et al., 2020; Hackney et al., 2007; Hackney and Earhart, 2009; Haas et al., 2024; Frisaldi et al., 2021; Feng et al., 2019; Duncan and Earhart, 2014; Duncan and Earhart, 2012; De Luca et al., 2020; de Bruin et al., 2010; Calabrò et al., 2019) exhibited a high risk of bias in the blinding of participants and personnel, with no studies at low risk.

D4: For blinding of outcome assessment, 24 studies (Volpe et al., 2013; Van Puymbroeck et al., 2018; Thaut et al., 2019; Son and Choi, 2018; Shanahan et al., 2017; Romenets et al., 2015; Pohl et al., 2020; Modugno et al., 2010; Lee et al., 2018; Kwok et al., 2019; Kunkel et al., 2017; Khuzema et al., 2020; Hackney et al., 2007; Hackney and Earhart, 2009; Haas et al., 2024; Frisaldi et al., 2021; Feng et al., 2019; Duncan and Earhart, 2014; Duncan and Earhart, 2012; De Luca et al., 2020; Calabrò et al., 2019) were assessed as having a low risk of bias (84.62%), with 11.54% showing high risk and 3.84% remaining unclear.

D5: In terms of incomplete outcome data, 20 studies (Volpe et al., 2013; Vitale et al., 2024; Van Puymbroeck et al., 2018; Son and Choi, 2018; Solla et al., 2019; Shanahan et al., 2017; Romenets et al., 2015; Pohl et al., 2020; Michels et al., 2018; Li et al., 2022; Kwok et al., 2019; Khuzema et al., 2020; Hackney et al., 2007; Hackney and Earhart, 2009; Haas et al., 2024; Frisaldi et al., 2021; Feng et al., 2019; Duncan and Earhart, 2014; Duncan and Earhart, 2012; De Luca et al., 2020; Calabrò et al., 2019) were found to have a low risk of bias (76.92%), while 23.08% were rated as high risk.

D6: Regarding selective reporting and outcome reporting, 28 studies (Volpe et al., 2013; Vitale et al., 2024; Van Puymbroeck et al., 2018; Thaut et al., 2019; Son and Choi, 2018; Solla et al., 2019; Shanahan et al., 2017; Romenets et al., 2015; Pohl et al., 2020; Modugno et al., 2010; Michels et al., 2018; Li et al., 2022; Lee et al., 2018; Kwok et al., 2019; Kunkel et al., 2017; Khuzema et al., 2020; Hackney et al., 2007; Hackney and Earhart, 2009; Haas et al., 2024; Frisaldi et al., 2021; Feng et al., 2019; Duncan and Earhart, 2014; Duncan and Earhart, 2012; De Luca et al., 2020; de Bruin et al., 2010; Calabrò et al., 2019) showed a low risk of bias, making up 100%.

D7: For other types of bias, 11 studies (Thaut et al., 2019; Romenets et al., 2015; Pohl et al., 2020; Li et al., 2022; Lee et al., 2018; Kwok et al., 2019; Frisaldi et al., 2021; Duncan and Earhart, 2012; De Luca et al., 2020; Calabrò et al., 2019) exhibited a low risk (38.46%), while 61.54% were found to have a high risk.

### 2.5 Data analysis

We conducted analyses using R software (version 4.1.2) with the “meta” package. For each intervention group, standardized mean differences (SMD) and their corresponding standard deviations were calculated. To compare various art therapies with control groups, pairwise meta-analyses were performed. Heterogeneity was assessed using the Cochrane Q statistic and I^2^ index, with thresholds indicating low (0–25%), moderate (25–50%), and substantial (>50%) heterogeneity. When heterogeneity exceeded 30%, a random-effects model was applied; otherwise, a common-effects model was used. Statistical significance was determined by a two-sided *p*-value of less than 0.05. Publication bias was assessed through Egger’s test and funnel plot visualization.

Setting a 30% threshold for heterogeneity assessment facilitates the early detection of variability across studies included in this meta-analysis. Compared to the conventional 50% threshold, the choice of a 30% cutoff enables the application of a random-effects model under moderate heterogeneity, thereby more comprehensively accounting for inter-study differences (Higgins et al., 2003). This approach improves the accuracy and robustness of the meta-analysis findings.

## 3 Results

### 3.1 Study identification

**Figure 2:**
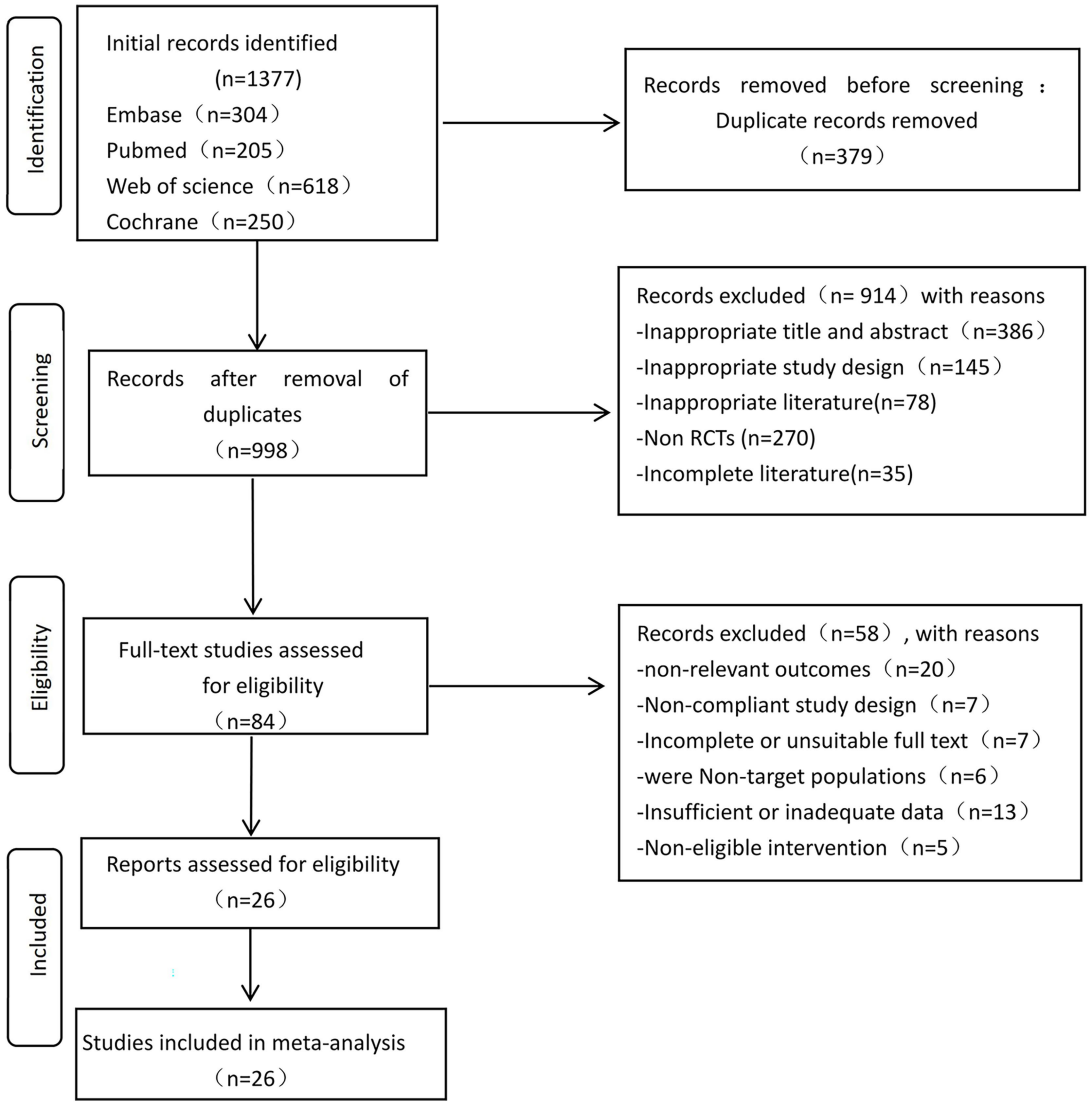
PRISMA flow diagram.

This search yielded 1,377 citations. After removing duplicates, 998 articles were left for screening. Of these, 914 were excluded due to issues with titles and abstracts, Non RCTs, Literature review, meta analyse, comments, Unrelated literature, Incomplete literature etc. The remaining 84 full−text articles were evaluated for eligibility; 20 were non-relevant outcomes, 7 were non-compliant study design, 7 were Incomplete or unsuitable full text, 6 were non-target populations, 13 Insufficient or inadequate data, 5 non-eligible intervention, Ultimately, 26 studies included in review (Figure 2).

### 3.2 Study characteristics

This meta-analysis incorporated 2,756 individuals from 26 randomized controlled trials. The studies were conducted between 2007 and 2024. All control groups were non-art therapy. The average age of participants in the experimental group was 65.98 ± 3.81 years, with 60.86% being male, and the mean disease duration was 4.01 ± 2.95 years. In the control group, the average age was 68.97 ± 6.08 years, with 51.98% being male, and the mean disease duration was 5.38 ± 3.38 years. 53.85% of the trials had a registration number. The studies included seven from Italy, seven from the United States one from Sweden, three from China (including Hong Kong), one from India, one from Brazil, one from Ireland, two from Korea, two from Canada, and one from the United Kingdom.

### 3.3 Characteristics of the intervention

Among the 26 studies, the types of art-based interventions were diverse. Specifically, 5 studies utilized music, 14 focused on dance, 2 employed yoga, 1 incorporated mindfulness, 1 used theater, 1 employed game-based therapy through virtual reality (VR), 1 combined Tai Chi and yoga, and 1 combined Qigong and dance. This variety demonstrates the wide range of creative and therapeutic approaches used in the included studies. Four were combined art therapies. Training sessions lasted between 30 and 120 min, with program durations ranging from 4 weeks to 3 years. Control interventions varied widely: 18 of them were passive control conditions, including two wait−list controls and 13 usual care or no intervention. Ten of them were active control conditions, including physical interventions such as physical therapy, walking, Nordic Walking, Deep Water swimming, podcasts, stretching, flexibility exercises, Stretching and Resistance Training Exercises, and multimodal exercise programs. The UPDRS III was used in 19 of the 26 studies and the TUG in 20 studies, reflecting their frequent utilization (Table 2).

**Table 2:** Summary table of included reviews.

Study	Study design	Country	Types of art	Sample size	Gender (M)	Mean age	Disease duration	Funding registration	Intervention		Outcome
				EG CG	EG CG	EG CG	EG CG		Intervention content Time, frequency, period (EG)	Intervention content Time, frequency, period (CG)	
Volpe 2013	RCT	Italy	Dance	EG:12 CG:12	EG: 58.33% CG: 50%	EG: 61.6±4.5 years CG: 65±5.3 years	EG: 9.0±3.6 years CG: 8.9±2.5 years	2012-005769-11	Irish set dancing classes (1.5-h sessions once a week for 6 months)	Physiotherapy (1.5-h sessions once a week for 6 months)	UPDRSIII, FOG
Vitale 2024	RCT	Italy	Dance	EG:14 CG:14	EG: 85.71% CG: 71.43%	EG: 64.1±7.9 years CG: 62.9±4.7 years	EG: 5.5±2.8 years CG: 6.5±3.5 years	N/A	Biodanza involves movement, music, and emotional experiences (2-h sessions once a week for 12 weeks)	No intervention or motor activity	UPDRSIII, TUG
Thaut 2019	RCT	United States	Music	EG:25 CG:23	EG: 68% CG: 68.18%	EG: 71 ± 7years CG; 73 ± 8years	EG: 10.9 ± 5 years CG: 11.2 ± 6 years	NCT03316365	Gait training with rhythmic auditory stimulation (RAS) (30 min per day, for 24 weeks)	Stopped training between weeks 8 and 16	Gait speed, stride length
Son 2018	RCT	Korea	Mindfulness	EG:33 CG:30	EG: 42.42% CG: 30%	EG; 63.2 ± 1.13 years CG: 62 ± 1.58 years	EG: 9.4 years CG: 10 years	N/A	Mindfulness meditation combined with physical exercises (2-h sessions, once a week, for 8 weeks)	No meditation or exercise intervention (routine outpatient care)	6MWT
Shanahan 2017	RCT	Ireland	Dance	EG:20 CG:21	EG: 65% CG: 61.90%	EG; 69 ± 10 years CG: 69 ± 8 years	EG: 5.5 ± 6 years CG: 6 ± 8 years	NCT01939717	Irish set dancing (1.5-h sessions once a week for 10 weeks)	No additional intervention, maintaining routine care and activities (usual care and daily activities)	6MWT, MINI BEST
Romenets 2015	RCT	Canada	Dance	EG:18 CG:15	EG; 66.67% CG: 46.67%	EG: 63.2 ± 9.9 years CG: 64.3 ± 8.1 years	EG: 5.5 ± 4.4 years CG: 7.7 ± 4.6 years	NCT01573260	Argentine tango classes (two 1-h sessions per week for 12 weeks)	Routine home exercises based on provided Parkinson’s disease exercise guidelines (daily self-guided exercise, no fixed time)	UPDRSIII, FOG, MINI BEST
Puymbroeck 2018	RCT	United States	Yoga	EG:15 CG:12	EG: 66.67% CG: 58.33%	EG: 65.53 ± 6.09 years CG: 70.5 ± 4.44 years	N/A	Pro00041068	Yoga training (two 2-h sessions per week for 8 weeks)	No intervention, only regular follow-up by phone (no intervention, only phone follow-ups)	UPDRSIII, FOG, MINI BEST
Pohl 2020	RCT	Sweden	Music	EG:23 CG:15	EG: 65.38% CG: 70%	EG: 69.7 ± 7.0years CG: 70.4 ± 6.0years	EG: 6.0 ± 4.4 years CG: 6.8 ± 3.6 years	NCT02999997	The Ronnie Gardiner Method, which combines multitasking exercises with visual symbols, coordinated body movements, and verbal synchronization to music rhythm (two 60-min sessions per week for 12 weeks)	Usual care with no additional activities (no additional intervention)	TUG, FOG, MINI BEST
Paolo 2019	RCT	Italy	Dance	EG:10 CG:9	EG: 60% CG: 70%	EG: 67.8 ± 5.9 years CG: 67.1 ± 6.3 years	EG: 4.4 ± 4.5 years CG: 5 ± 2.9 years	NP/3339	Traditional Sardinian folk dance (two 90-min sessions per week for 12 weeks)	No additional intervention, continuing with regular medical care (no additional intervention)	6MWT, TUG, gait speed, stride length
Modugno 2010	RCT	Italy	Theater	EG:10 CG:10	EG: 60% CG: 50%	EG: 63 ± 1.13 years CG: 62 ± 1.58 years	EG: 9.4 ± 4.5 years CG: 10 ± 5 years	N/A	The theater workshops (one or two workshops per month, 6 h each, over a period of 3 years)	Physical therapy included muscle stiffness prevention, posture correction, balance training, and gait training (three weekly sessions, 2–3 h each, over 3 years)	UPDRSIII
Michels 2018	RCT	United States	Dance	EG:9 CG:4	EG: 66.67% CG: 25%	EG: 66.44 years CG: 75.5 years	EG: 2.11 ± 0.33 years CG: 2.50 ± 1.00 years	N/A.	Dance therapy (60-min sessions once a week for 10 weeks)	The support group involved education and emotional support discussions without any physical activity (weekly discussions and education sessions without physical activity)	UPDRSIII, TUG
Luca 2020	RCT	Italy	Music	EG:20 CG:20	EG: 50% CG: 35%	EG: 63.2 ± 8.4 years CG: 66.5 ± 6.2 years	EG: 9.3 ± 4.8 years CG: 10.1 ± 5.2 years	N/A.	Rhythmic music beat (3 sessions per week, 30 min each, for 8 weeks)	Conventional gait training without music assistance or real-time gait feedback (3 sessions per week, 30 min each, for 8 weeks)	TUG
Li 2022	RCT	China	Dance Music	EG:17 EG1:18 CG:14	EG:50% EG1:50% CG:46.67%	EG: 67.40 ± 6.06 years EG1:69.16 ± 6.17 years CG:67.18 ± 7.2 years	EG:3.65 ± 3.37 years EG1:4.89 ± 3.53 years CG:25.03 ± 3.66 years	ChiCTR2200061252	(1) Yang-ge dancing, participants practiced with props (fan and handkerchief) to music, (2) Conventional exercise with music (60 min per session, 5 sessions per week, for 4 weeks)	CG: Conventional exercise, EG1: 5 sessions per week, for 4 weeks	UPDRSIII
Lee 2018	RCT	Korea	Dance Qigong	EG:25 CG:16	EG: 40% CG: 43.75%	EG: 65.8 ± 7.2 years CG: 65.7 ± 6.4 years	EG: 4.5 ± 3.3 years CG: 4.4 ± 3.0 years	IRB No.200902	Turo PD, a hybrid qigong-dance program (60 min per session, twice a week, for 8 weeks)	No intervention during the 8-week period, followed by participation in the same Turo PD program (N/A during the first 8 weeks, then the same program as the Turo PD group)	UPDRSIII
Kwok2019	RCT	Hong Kong, China	Yoga	EG:57 CG:55	EG: 52.1% CG: 41.8%	EG: 63.7 ± 8.2 years CG: 63.5 ± 9.3 years	N/A	CUHK_CCRB00522	Mindfulness Yoga (90-min sessions, once a week, for 8 weeks)	SRTE, consisting of 60-min group sessions focusing on stretching, resistance training, and warm-up exercises (60-min sessions, once a week, for 8 weeks)	UPDRSIII, TUG
Kunkel 2017	RCT	United Kingdom	Dance	EG:31 CG:15	EG: 52.78% CG: 40%	EG: 71.3 ± 7.7 years CG: 69.7 ± 6.0 years	EG: 4.7 ± 3.5 years CG: 7.0 ± 4.9 years	ISRCTN 63088686	Ballroom dancing sessions (twice a week for 10 weeks)	Control group participants received usual care and were offered dance class vouchers at the end of the study (No intervention during the trial period, usual care during the trial period, usual care)	TUG, 6MWT
Khuzema 2020	RCT	India	TaiChi, Yoga	EG:9 EG1:9 CG:9	EG: 66.67% EG1: 66.67% CG: 77.78%	EG:72 ± 5.22 years EG1:68.11 ± 4.23 years CG:70.89 ± 6.01 years	EG:5.67 ± 2.33 years EG1: 6.2 ± 1.67 years CG:5.23 ± 3.12 years	N/A	EG: Home-based Tai Chi exercises. EG1: Home-based Yoga exercises (30–40 min per session, 5 days a week, for 8 weeks)	Conventional balance exercises like backward walking and trunk rotations (40–45 min per session, 5 days a week, for 8 weeks)	TUG
Hackney 2009	RCT	United States	Dance	EG:14 EG1:17 CG:17	EG:78.57% EG1:64.71% CG:70.59%	EG: 68.2 ± 1.4 years EG1:66.8 ± 2.4 years CG:66.5 ± 2.8 years	EG: 6.9 ± 1.3 years EG: 9.2 ± 1.5 years CG:5.9 ± 1.0 years	N/A	EG: Argentine Tango dance classes. EG1: American Waltz and Foxtrot dance classes [1-h sessions, twice per week, for 13 weeks (20 lessons total)]	No intervention (no sessions during the study period)	UPDRSIII, TUG, 6MWT, FOG
Hackney 2007	RCT	United States	Dance	EG:9 CG:10	EG: 66.67% CG: 60%	EG: 72.6 ± 2.2 years CG: 69.6 ± 2.1 years	EG: 6.2 ± 1.5 years CG: 3.3 ± 0.5 years	N/A	Tango dance lessons (1-h sessions, twice a week, for 13 weeks)	Participants engaged in chair-based strength and flexibility exercises with some standing exercises (1-h sessions, twice a week, for 13 weeks)	UPDRSIII, TUG, FOG
Haas 2023	RCT	Brazil	Dance	EG:31 CG:21 CG1:31	EG:67.74% CG:19.05% CG1:25.81%	EG: 71.6 ± 8.9 years CG: 66.8 ± 9.0 years CG1:67.9 ± 11.2 years	EG: 5.6 ± 5.1 years CG: 8.0 ± 4.7 years CG1:7.0 ± 5.1 years	NCT03370315	Brazilian dance (60-min sessions, twice a week for 12 weeks)	CG: Deep-water running and muscle strength exercises. CG1: Nordic walking focusing on coordination and balance with walking poles (60-min sessions, twice a week for 12 weeks)	UPDRSIII, TUG, 6MWT
Frisaldi 2021	RCT	Italy	Dance	EG:19 CG:19	EG: 52.63% CG: 68.42%	EG: 60.68 ± 6.34years CG: 61.21 ± 7.18years	EG: 5.99 ± 2.18 years CG: 6.43 ± 2.50 years	N/A	1 h of conventional physiotherapy followed by 1 hour of dance class (focused on contemporary dance and ballet without music) (1 h of physiotherapy and 1 h of dance, 3 times a week, for 5 weeks)	Physiotherapy followed by another hour of conventional physiotherapy (1 h of physiotherapy, 3 times a week, for 5 weeks)	UPDRSIII, TUG, 6MWT, FOG, MINI BEST
Feng 2019	RCT	China	Game (VR)	EG:14 CG:14	EG: 53.33% CG: 60%	EG: 67.47 ± 4.79years CG; 66.93 ± 4.64years	EG: 7.07 ± 1.44 years CG: 6.60 ± 1.45 years	N/A	Virtual reality (VR) training (45 min per session, 5 days a week, for 12 weeks)	Conventional physical therapy following the 2014 edition of the Chinese Guide to Parkinson’s Disease treatment (45 min per session, 5 days a week, for 12 weeks)	TUG
Duncan 2014	RCT	United States	Dance	EG:5 CG:5	EG: 80% CG: 80%	EG: 69.6 ± 6.6 years CG: 66 ± 11.0 years	EG: 6.6 ± 7.5 years CG: 11 ± 3.9 years	N/A	Argentine tango (AT) dance classes (1-h session, twice weekly, for 2 years)	No prescribed exercise	UPDRSIII, TUG, 6MWT, FOG, MINI BEST
Duncan 2012	RCT	United States	Dance	EG:26 CG:26	EG; 57.69% CG: 57.69%	EG: 69.3 ± 1.9 years CG: 69.0 ± 1.5 years	EG: 5.8 ± 1.1 years CG: 7.0 ± 1.0 years	NCT01388556	Community-based Argentine Tango classes (1-h session, twice weekly, for 12 months)	No prescribed exercise	UPDRSIII, 6MWT, FOG, MINI BEST
Calabrò 2019	RCT	Italy	Music	EG:25 CG:25	EG; 45% CG: 30%	EG: 70 ± 8 years CG: 73 ± 8 years	EG: 10 ± 3 years CG: 9.3 ± 3 years	NCT03434496	Treadmill gait training without Rhythmic Auditory Stimulation (non-RAS) (30 min per day, 5 times per week, for 8 weeks)	Treadmill gait training without Rhythmic Auditory Stimulation (non-RAS) (30 min per day, 5 times per week, for 8 weeks)	TUG, Gait speed, Stride length
Bruin 2010	RCT	Canada	Music	EG:11 CG:11	EG: 54.55% CG: 45.45%	EG: 64.1 ± 4.2 years CG: 67 ± 8.1years	EG: 6.4 ± 4.2years CG: 4.5 ± 3.3years	N/A	Walking while listening to -matched, individualized music playlists (30 min per session, 3 times per week, for 13 weeks)	Continued with regular activities (no music intervention) (regular activities without specific intervention for 13 weeks)	UPDRSIII, Gait speed, Stride length

### 3.4 Effects of art therapy on Parkinson’s disease

**Meta-analysis of the difference**: Given the significant differences between pre − and post−intervention, this study conducted a meta−analysis using SMD. This method directly captures the intervention’s effect while controlling for baseline differences, improving the accuracy and statistical power (Tables 3, 4).

**Table 3:** Meta analysis of baseline evaluation at enrolling.

Outcome	No. of study	No. of individuals	Model	SMD (95%CI)	*P* value	*I* ^2^	P for heterogeneity
UPDRGIII	19	685	Fixed	0.06 [−0.09; 0.21]	0.45	0%	0.49
6MWT	11	453	Fixed	−0.15 [−0.33; 0.04]	0.123	0%	0.99
FOG	10	309	Fixed	0.29 [0.06; 0.51]	0.013	0%	0.86
GIAT SPEED	5	161	Random	0.23 [−0.27; 0.73]	0.369	62%	0.03
MINI BEST	7	245	Fixed	0.09 [−0.16; 0.34]	0.48	0%	0.53
STRIDE LENGTH	5	161	Fixed	−0.06 [−0.37; 0.26]	0.73	6%	0.37
TUG	21	706	Random	0.16 [−0.06; 0.37]	0.149	45%	0.02

We first compared baseline questionnaire scores in individuals before intervention. Individuals receiving art therapies has similar UPDRGIII (*P* = 0.458), TUG (*P* = 0.149), 6-minute test (6MWT) (*P* = 0.123), GAIT SPEED (*P* = 0.369), stride length (*P* = 0.73) and MINI BEST (*P* = 0.48) at enrollment compared to those in the control groups. Risk of bias.

**Table 4:** Meta-analysis of post-intervention evaluation at the end.

Outcome	No. of study	No. of individuals	Model	SMD (95%CI)	*P* value	*I* ^2^	P for heterogeneity
UPDRGIII	19	685	Random	−0.56 [−0.88; −0.25]	0.0001	70%	*P* < 0.01
6MWT	11	453	Random	0.63 [−0.01; 1.27]	0.053	87%	*P* < 0.01
FOG	10	309	Random	−0.04 [−0.43; 0.35]	0.85	60%	*P* < 0.01
GIAT SPEED	5	161	Fixed	0.62 [0.30; 0.94]	0.0001	0%	0.56
MINI BEST	7	245	Random	0.49 [0.11; 0.87]	0.01	50%	0.06
STRIDE LENGTH	5	161	Fixed	−0.06 [−0.37; 0.26]	0.0001	6%	0.37
TUG	21	706	Random	−0.13 [−0.44; 0.18]	0.34	69%	*P* < 0.01

Further, we evaluated treatment effects after intervention. Individuals receiving art therapies had UPDRGIII (SMD= −0.56, 95%CI [−0.88, −0.25], *P* < 0.05), 6MWT (SMD = 0.63, 95%CI [−0.01, −1.27], *P* = 0.053), FOG (SMD= −0.04, 95%CI [−0.43, 0.35], *P* > 0.05), Gait speed (SMD = −0.62, 95%CI [0.3, 0.94], *P* < 0.05), MINI Best (SMD = 0.49, 95%CI [0.11, 0.87], *P* < 0.05), Stride length (SMD = −0.06, 95%CI [−0.37, 0.26], *P* < 0.05), TUG (SMD = −0.13 95%CI [−0.44, 0.18], *P* > 0.05).

Effect sizes are interpreted based on Cohen’s guidelines, defining small (SMD = 0.2), medium (SMD = 0.5), and large (SMD = 0.8) effects, and are further refined by relevant meta−analytic literature: Small Effect 0.2 ≤ SMD < 0.35, Slightly Medium Effect 0.35 ≤ SMD < 0.5, Medium Effect 0.5 ≤ SMD < 0.65, Slightly Large Effect 0.65 ≤ SMD < 0.8, Large Effect SMD ≥ 0.8.

#### 3.4.1 Primary outcomes

##### 3.4.1.1 UPDRS III

**Figure 3:**
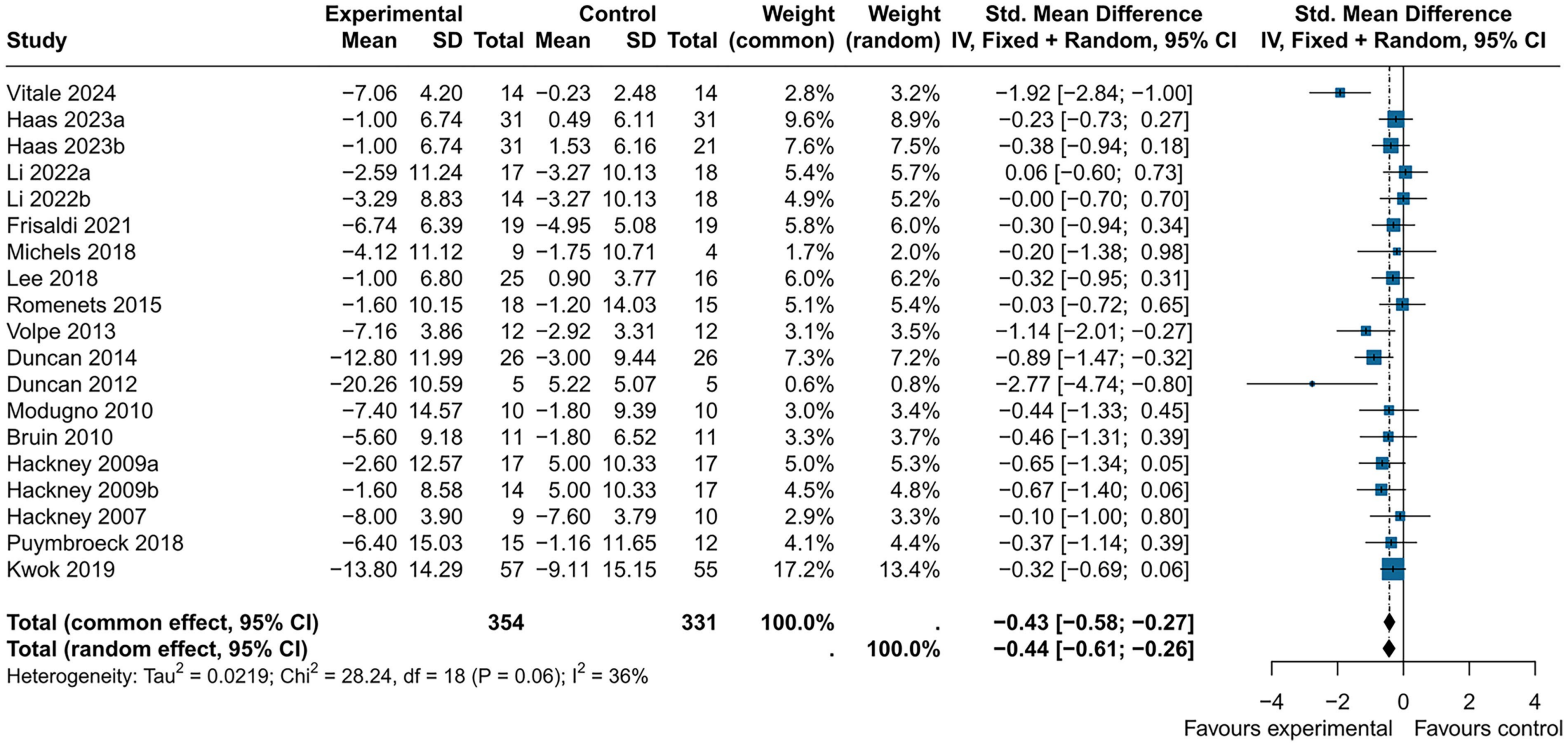
Forest plot of the UPDRS part III.

**Figure 4:**
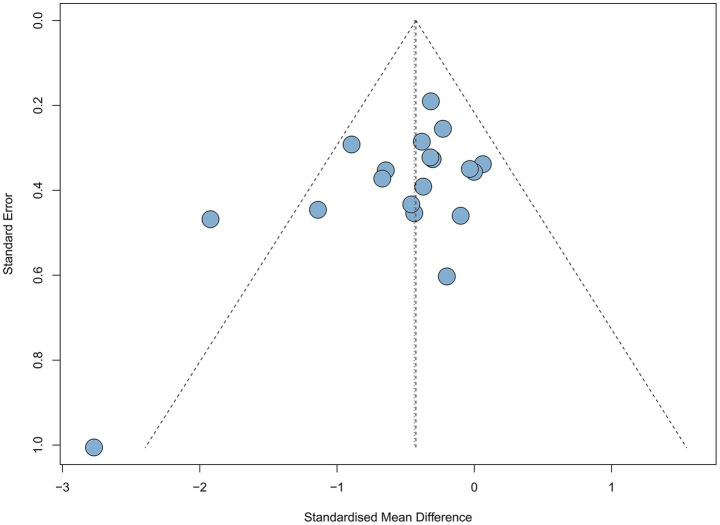
Funnel plot of UPDRS part III.

UPDRS III is used to measure motor function in PD patients, with lower scores after intervention indicating better motor function. Nineteen studies (*n* = 685). utilized the UPDRS III to assess treatment effects. Revealing a significant reduction in UPDRS III scores in the experimental group compared to the control group (SMD = −0.44, 95% CI [−0.61; −0.26], *p* < 0.05), with medium effect and statistical significance, with high heterogeneity (*I*^2^ = 36%) (Figure 3). Excluding the study by Vitale. reduced heterogeneity (*I*^2^ = 4%). Egger’s test indicated no publication bias (*p* = 0.07). Although the results were statistical significance, the high heterogeneity, possibly due to the small sample size, and the bias risk identified by Egger’s test suggest that these findings should be interpreted with caution. Which was further supported by the funnel plot (Figure 4).

**Figure 5:**
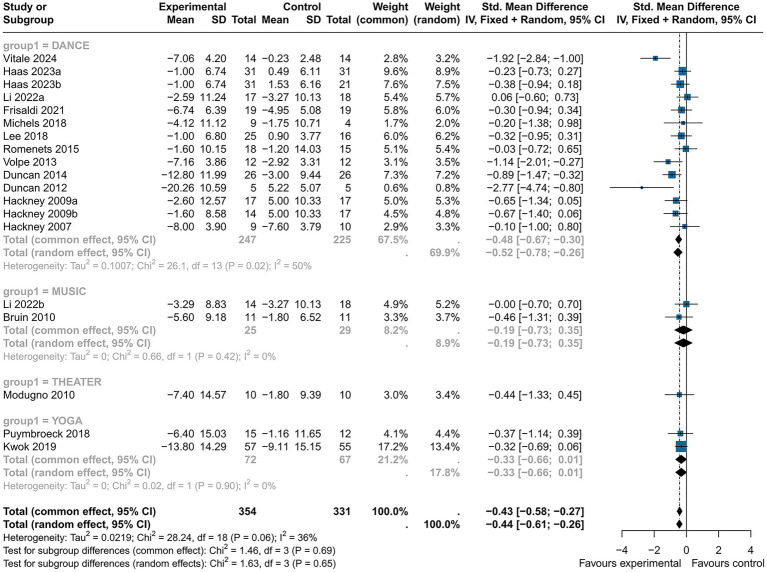
The subgroup analysis of the effect of art therapy intervention types of UPDRS part III.

Subgroup analyses indicated that music (SMD = −0.19, 95% CI [−0.73; 0.35], *p* > 0.05), theater (SMD = −0.44, 95% CI [−1.33; 0.45], *p* > 0.05) and yoga intervention (SMD = −0.33, 95% CI [−0.66; −0.01], *p* > 0.05) were non-significant. Dance intervention (SMD = −0.52, 95% CI [−0.78; −0.26], *p* < 0.05) showed a medium effect and statistical significance, but with high heterogeneity (*I*^2^ = 50%) (Figure 5).

##### 3.4.1.2 TUG

**Figure 6:**
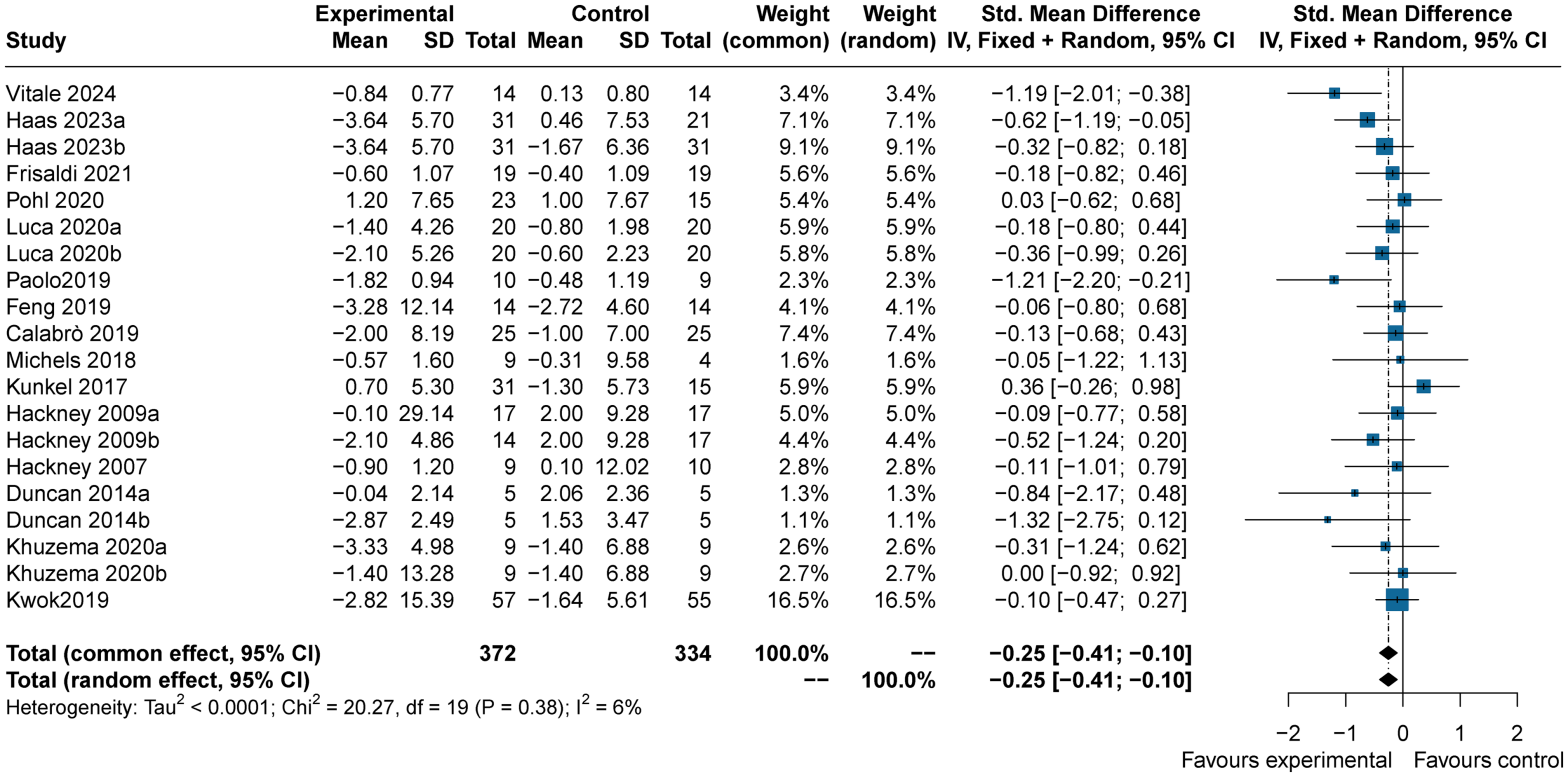
Forest plot of the TUG.

Twenty studies (*n* = 742) reported the effects of art therapy interventions on TUG scores. The TUG test is widely used to evaluate dynamic balance, gait function in PD patients. Results demonstrated a reduction in TUG scores in the experimental group compared to the control group (SMD = −0.25, 95% CI [−0.41, −0.10], *p* < 0.05), indicating a small effect size with low heterogeneity (*I*^2^ = 6%) (Figure 6). Egger’s test showed no evidence of publication bias (*p* = 0.08). These findings suggest art therapy has a modest but statistically significant impact on dynamic balance and gait function.

**Figure 7:**
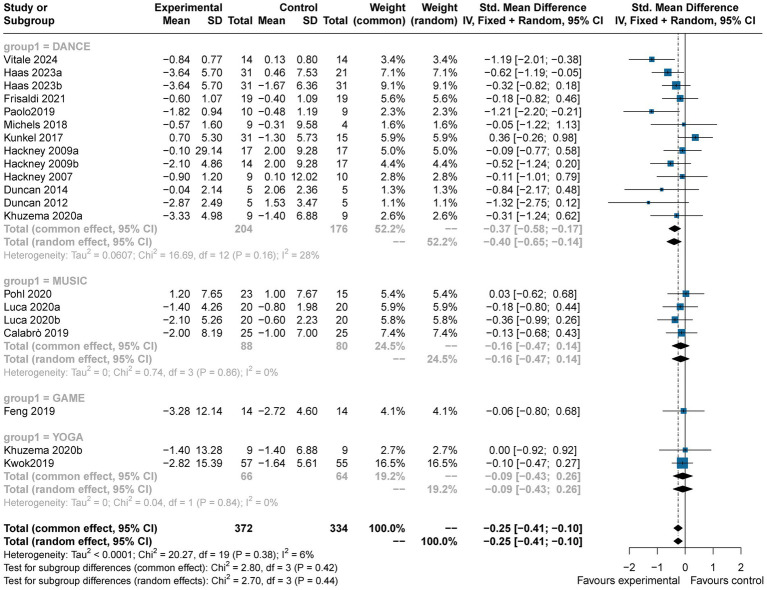
The subgroup analysis of the effect of art therapy intervention types of TUGS.

Subgroup analysis showed that music (SMD = −0.16, 95% CI [−0.47, 0.14], *p* > 0.05), game-based interventions (SMD = −0.06, 95% CI [−0.80, 0.68], *p* > 0.05), and yoga (SMD = −0.09, 95% CI [−0.43, 0.26], *p* > 0.05) yielded small, non-significant effects. However, dance interventions resulted in a greater reduction in TUG scores (SMD = −0.37, 95% CI [−0.58, −0.17], *p* < 0.05), with a slightly medium effect size and moderate heterogeneity (*I*^2^ = 28%). This underscores the potential of dance therapy to improve motor function in PD patients (Figure 7).

##### 3.4.1.3 Mini–BESTest

**Figure 8:**
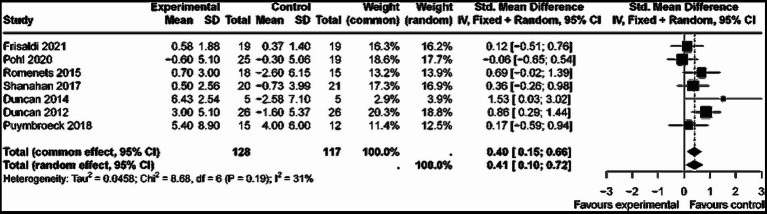
Forest plot of the Mini−BESTest.

The Mini–BESTest, used to assess balance functions (e.g., postural control and gait stability), showed that art therapy participants had higher scores (SMD = 0.41, 95% CI [0.10; 0.72], *p* < 0.05) across seven studies (*n* = 245), with moderate heterogeneity (*I*^2^ = 31%) reduced to 9% upon excluding (Duncan, 2012) (Figure 8). Publication bias was not found (*p* = 0.356). Art therapy showed a slightly medium effect and statistical significance.

**Figure 9:**
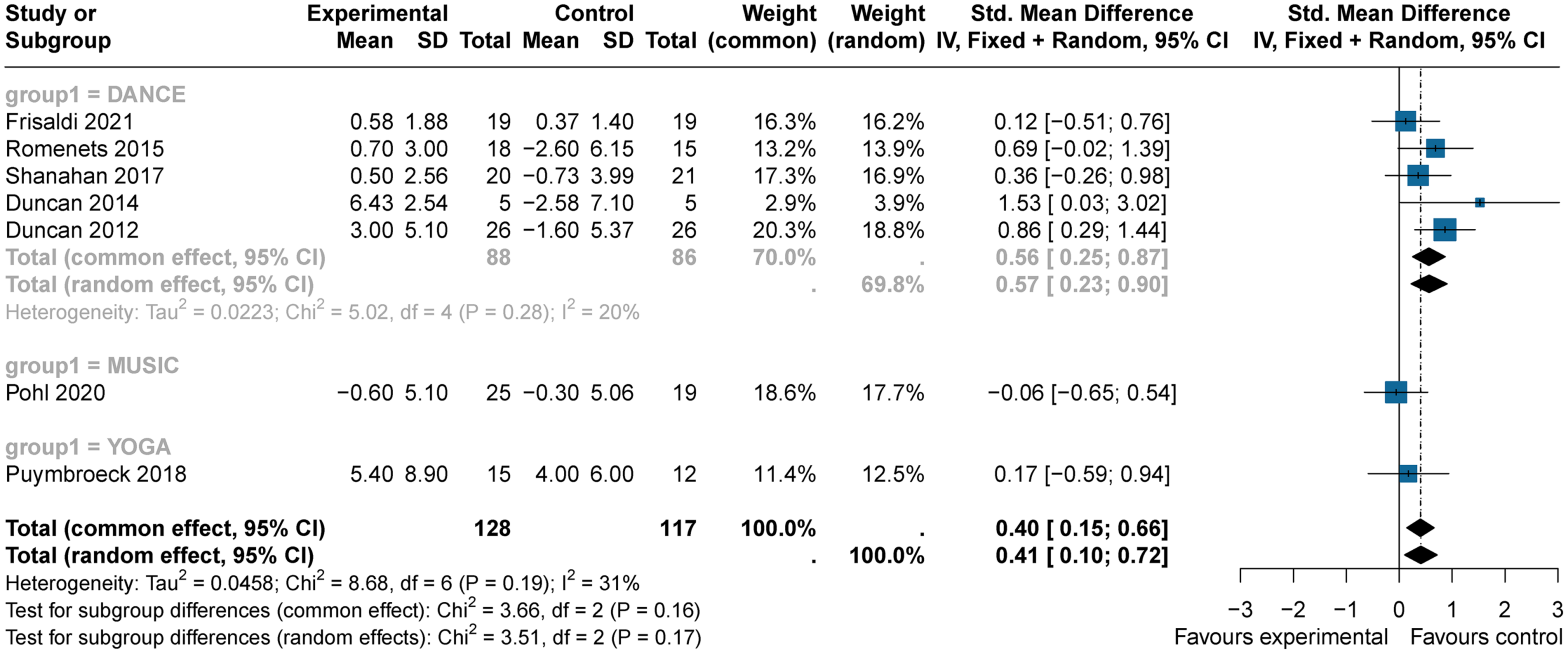
The subgroup analysis of the effect of art therapy intervention types on Mini−BESTest.

Subgroup analysis indicated that music (SMD = −0.06, 95% CI [−0.65; 0.54], *p* > 0.05) and yoga (SMD = 0.17, 95% CI [−0.59; 0.94], *p* > 0.05) were non-significant, while dance showed the strongest effect (SMD = 0.56, 95% CI [0.25, 0.87], *p* < 0.05) with low heterogeneity (*I*^2^ = 20%). This medium effect underscores dance as the most effective intervention with statistical significance (Figure 9).

#### 3.4.2 Secondary outcomes

##### 3.4.2.1 Gait speed

Gait speed, an indicator of walking performance, was assessed in five studies (*n* = 161). Art therapy participants demonstrated improved gait speed scores compared to controls (SMD = 0.34, 95% CI [0.03, 0.65], *p* < 0.05), with low heterogeneity (*I^2^* = 0%) and no evidence of publication bias (*p* = 0.9), suggesting a small but statistically significant effect.

##### 3.4.2.2 6MWT

The 6MWT, a measure of cardiovascular endurance and functional capacity, was evaluated in 11 studies (*n* = 453). Art therapy participants demonstrated higher 6MWT scores compared to controls (SMD = 0.41, 95% CI [0.11, 0.72], *p* < 0.05), indicating a slightly medium effect size with statistical significance. However, high heterogeneity was observed (*I*^2^ = 60%), which decreased to moderate levels (*I*^2^ = 43%) after excluding the study by Paolo et al. Publication bias was detected (*p* = 0.04).

##### 3.4.2.3 FOG

The FOG scale, which assesses the severity and frequency of freezing episodes during walking, was evaluated in 10 studies (*n* = 309). Art therapy interventions demonstrated a small but statistically significant reduction in FOG scores compared to controls (SMD = −0.33, 95% CI [−0.58, −0.09], *p* < 0.05), with moderate heterogeneity (*I*^2^ = 32%) and no evidence of publication bias (*p* = 0.36). Excluding the study by Volpe reduced heterogeneity to 0%, further supporting the robustness of the results.

##### 3.4.2.4 Stride length

Stride length, a measure of gait and walking ability, reflects improvements in walking efficiency, balance, and lower limb function when increased. Five studies (*n* = 161) reported a medium effect size for art therapy interventions (SMD = 0.59, 95% CI [0.09, 1.09], *p* < 0.05), indicating statistical significance. Moderate heterogeneity was observed (*I*^2^ = 57%) with no evidence of publication bias (*p* = 0.61). Excluding the Thaut’s study reduced heterogeneity to 0%, but subsequent analysis revealed no statistically significant difference in stride length between art therapy and conventional treatments. Sequential exclusion of the Paolo and Calabrò’s studies did not significantly alter heterogeneity, but reversed the findings, showing no statistical significance.

## 4 Discussion

PD significantly affects motor function and gait, both of which are essential for daily activities and independent living. As the disease progresses, impairments such as freezing of gait and reduced stride length lead to mobility loss, worsening disability, and diminishing quality of life (Noyes et al., 2006). Globally, PD prevalence has doubled over the past 25 years, with over 8.5 million cases reported in 2019. PD also accounts for the fastest-growing rates of disability and mortality among neurological disorders, with 5.8 million disability-adjusted life years (DALYs) and 329,000 deaths in 2019, representing increases of 81% and over 100%, respectively, since 2000 (World Health Organization, 2022).

Improving motor function and gait is critical to mitigating PD’s functional decline, enhancing independence, and reducing daily risks. However, current treatments are expensive, do not cure the disease, and often carry significant side effects. Medications like levodopa (L-Dopa), while initially effective, lose their therapeutic efficacy over time. They can also cause nausea, hypotension, L-Dopa-induced dyskinesia, and motor fluctuations, while COMT inhibitors may lead to gastrointestinal issues and, in severe cases, fatal liver toxicity. Surgical treatments such as deep brain stimulation (DBS) pose risks of infection, bleeding, cognitive decline, and speech or balance disorders. Despite these interventions, all available treatments fail to cure the disease, and PD remains a progressive disorder that ultimately leads to severe disability—particularly due to increasingly treatment-resistant motor and gait impairments, which are critical to maintaining functional independence (Poewe et al., 2017). Given the significant limitations and adverse effects of existing treatments for PD, exploring alternative therapeutic approaches has become imperative. Studies in neuroaesthetics show that art therapy engages prefrontal–limbic integration, enhances default mode network (DMN) connectivity for flow states, modulates cortisol and dopamine levels, promotes empathy via mirror neuron activation, and facilitates theta–gamma coupling—together mediating neuroplastic therapeutic effects. Art therapy holds significant promise in addressing both motor and non-motor symptoms in individuals with Parkinson’s disease (PD). This therapeutic potential is supported by evidence suggesting that art therapy can enhance neuroplasticity and activate dopaminergic pathways, thereby improving emotional regulation. These neural adaptations may help mitigate psychosocial stress and psychosomatic symptoms, highlighting the therapeutic promise of art-based interventions through well-defined neurobiological mechanisms (Lauring et al., 2019). This meta-analysis focuses on evaluating the impact of art therapy on motor function, particularly gait and dynamic balance, which are crucial for maintaining independence and reducing the risk of complications. By analysing validated studies, this research sheds light on the therapeutic potential of art therapy, especially dance, in enhancing physical function in PD patients. Dance therapy integrates rhythmic movement, music, and social engagement, enhancing motor coordination, neuroplasticity, and dopamine release while stimulating sensorimotor integration, emotional regulation, and cognitive-motor synchronization to mitigate Parkinson’s symptoms.

The motor function of this meta-analysis was defined as a multidimensional concept encompassing both gait and dynamic balance, with gait serving as a core component. Relevant studies that used validated scales to measure these aspects were included to ensure a comprehensive evaluation. This study demonstrates that art therapy significantly improves both motor and gait functions in patients with PD. Among the specific types of art therapy interventions evaluated, including drama, music, yoga, and dance, dance consistently demonstrated the most remarkable effects. The use of multifunctional assessment scales provided a comprehensive evaluation of body function, enabling simultaneous analysis of gait and dynamic balance-two closely interconnected components of motor performance. This meta-analysis presents strong evidence that art therapy enhances physical function in PD patients, with dance interventions yielding the most substantial benefits. Specifically, dance showed the strongest effects on gait and dynamic balance, with a moderate effect size and low heterogeneity. Furthermore, dance interventions also demonstrated significant improvements in overall body function. These findings underscore the exceptional value of dance as a targeted intervention for improving gait and dynamic balance, offering promising clinical implications for PD rehabilitation.

Furthermore, the meta-analysis revealed significant overall improvements in secondary outcomes, including stride length, freezing of gait (FOG), the 6-min walk test (6MWT), and gait speed, following art therapy interventions. However, subgroup analyses were not conducted for these measures due to limited data availability, high heterogeneity in study designs and outcome measurements, and insufficient statistical power. These findings underscore the importance of future research with larger sample sizes, standardized protocols, and comprehensive data reporting to better explore subgroup-specific effects on secondary outcomes and provide more robust evidence for the role of art therapy in PD rehabilitation.

Studies demonstrate that art-based interventions significantly benefit motor function in Parkinson’s disease (PD). For instance, the study by Frisaldi et al. (2021) and Li et al. (2022) reported enhanced upper limb mobility using the DArT method and improved lower limb strength with Brazilian Dance Group (BDG) exercises. Similarly, Volpe et al. (2013) found that Irish dance, characterized by rhythmic music and large-amplitude movements, effectively addressed gait freezing, balance issues, and motor impairments. Hackney’s study (Hackney et al., 2007; Hackney and Earhart, 2009) showed that tango specifically targeted motor deficits related to gait and balance. de Bruin et al. (2010) and Puymbroeck’s studies (Van Puymbroeck et al., 2018) demonstrated the efficacy of music-assisted gait training and yoga in improving stride length, speed, rhythm, postural stability, and alleviating gait freezing. These findings suggest that rhythm-based therapies hold significant potential for managing motor symptoms, particularly in areas like gait and balance.

Art therapy also shows promise in addressing non-motor symptoms in PD. Pohl’s study suggests that group music interventions positively impact mental health and social engagement, though with minimal cognitive effects. Modugno et al. (2010) reported that combining active theater with conventional treatments enhanced cognitive, emotional, and motor functions, ultimately improving quality of life. Similarly, Kwok et al. (2019) highlighted that mindfulness yoga reduced anxiety and depression while enhancing motor function, mental health, and overall quality of life. Vitale et al. (2024) further demonstrated that Biodanza, an intervention combining movement, music, and group interaction, not only improved motor function but also strengthened cognitive and social outcomes. Despite these findings, some studies report that cognitive benefits and reductions in depressive symptoms often lack statistical significance, likely due to variations in sample size, intervention duration, or assessment sensitivity. This underscores the need for further research to refine art therapy protocols aimed specifically at cognitive outcomes.

Although the primary focus of this paper is on motor function, the long-term efficacy of art therapy in managing PD remains underexplored. Duncan’s longitudinal study (Duncan and Earhart, 2012) revealed that community dance programs could slow the progression of both motor and non-motor symptoms over 2 years, improving daily living activities and balance. However, challenges persist in evaluating the long-term effects of different art forms, particularly in mood and cognition. Volpe et al. (2013) noted the need for more studies quantifying the effects of various dance forms, such as modern dance, tango, and jazz ballet. Thaut et al. (2019) similarly emphasized the importance of exploring the sustained benefits of Rhythmic Auditory Stimulation (RAS) on non-motor symptoms.

Visual arts and painting, however, represent a significant research gap. While some studies examined Visual arts and painting as a tool for assessing disease progression (Kuosmanen et al., 2019; Celik et al., 2024), its direct therapeutic effects remain unexplored. This underscores the need for future research on the potential of visual arts, such as painting, in addressing non-motor symptoms like cognition and emotional health.

## 5 Future research directions

Emerging therapies such as clay therapy, game therapy, and virtual reality (VR) therapy also show promise. Although these fields are in their infancy and lack robust RCT data, integrating VR into therapeutic frameworks may offer innovative approaches to PD treatment. Future studies should explore these possibilities to enhance the scope of art-based interventions for PD.

## 6 Limitations

Dependence on secondary data from previously published RCTs introduces potential biases related to study design and reporting. Heterogeneity across studies—particularly in intervention types, sample sizes, and outcome measures—limits the consistency of conclusions. Notably, variability in art therapy implementation (session frequency, duration, and artistic mediums) creates challenges for cross-study synthesis. Additionally, the lack of participant and personnel blinding in some studies increases the risk of bias. The exclusion of non-English publications may introduce language bias in evidence selection; reliance on self-reported outcomes could lead to subjectivity, and the long-term sustainability of interventions remains uncertain without extended follow-up data. Future research should focus on larger, high−quality RCTs with standardized protocols and direct comparisons between different art therapy modalities.

## 7 Conclusion

This meta-analysis demonstrates that art therapy significantly improves motor and gait functions in patients with PD, offering robust evidence for its clinical utility. Motor function benefits were substantiated by reductions in UPDRS III and TUG scores and increases in Mini-BESTest scores. Additionally, secondary outcomes revealed small to moderate improvements in gait speed, 6MWT performance, FOG scores, and stride length, indicating a comprehensive impact on mobility and functional independence.

Notably, dance therapy exhibited the most pronounced effects across motor function outcomes, emphasizing its potential as a cornerstone intervention in multidisciplinary neurorehabilitation. Despite these promising results, the high heterogeneity observed in certain outcomes underscores the critical need for standardized protocols to ensure consistency, reproducibility, and broader applicability. Future research should focus on optimizing intervention designs, identifying patient-specific predictors of success, and exploring long-term benefits to maximize the therapeutic impact of art-based interventions for PD. These findings provide a compelling case for the integration of evidence-based art therapies, particularly dance therapy, into tailored treatment regimens aimed at enhancing quality of life and functional outcomes in PD populations.

## Data Availability

The original contributions presented in the study are included in the article/supplementary material, further inquiries can be directed to the corresponding author.
